# An Adaptive Protection System for Sensor Networks Based on Analysis of Neighboring Nodes

**DOI:** 10.3390/s21186116

**Published:** 2021-09-12

**Authors:** Ján Gamec, Elena Basan, Alexandr Basan, Alexey Nekrasov, Colin Fidge, Nikita Sushkin

**Affiliations:** 1 Faculty of Electrical Engineering and Informatics, Technical University of Košice, Letná 9, 04200 Košice, Slovakia; jan.gamec@tuke.sk; 2 Institute for Computer Technologies and Information Security, Southern Federal University, Chekhova 2, 347922 Taganrog, Russia; basanalex@gmail.com (A.B.); alexei-nekrassov@mail.ru (A.N.); nikita.sushkin@mail.ru (N.S.); 3 Faculty of Science, Queensland University of Technology (QUT), Gardens Point Campus, Brisbane, QLD 4001, Australia; c.fidge@qut.edu.au

**Keywords:** attacks, entropy, security, anomaly, detection, normalization, divergence, smart sensors

## Abstract

Creation and operation of sensor systems is a complex challenge not only for industrial and military purposes but also for consumer services (“smart city”, “smart home”) and other applications such as agriculture (“smart farm”, “smart greenhouse”). The use of such systems gives a positive economic effect and provides additional benefits from various points of view. At the same time, due to a large number of threats and challenges to cyber security, it is necessary to detect attacks on sensor systems in a timely manner. Here we present an anomaly detection method in which sensor nodes observe their neighbors and detect obvious deviations in their behavior. In this way, the community of neighboring nodes works collectively to protect one another. The nodes record only those parameters and attributes that are inherent in any node. Regardless of the node’s functionality, such parameters include the amount of traffic passing through the node, its Central Processing Unit (CPU) load, as well as the presence and number of packets dropped by the node. Our method’s main goal is to implement protection against the active influence of an internal attacker on the whole sensor network. We present the anomaly detection method, a dataset collection strategy, and experimental results that show how different types of attacks can be distinguished in the data produced by the nodes.

## 1. Introduction

Sensor systems and networks are being implemented in various spheres of human activity. Smart sensors are used to build Internet-of-Things systems, groups of mobile robots, smart cars, and so on [1]. In the process of creating and operating these systems, cyber security becomes a critical concern [2]. Because sensor systems are built on new architectural solutions and principles, they are characterized by new cyber security threats [3]. For example, clustering is often used to build sensory systems and occasionally a new leader is chosen for a cluster. Introducing an intruder into such a scheme can lead to the intruder itself becoming the leader of the cluster or can affect the division of nodes into groups. As a result, not only information flows and processes can be disrupted, but the physical environment can be affected. This problem is especially relevant for cyber-physical systems that manage physical objects or assets [4].

Many proposals to protect sensor networks include the use of Blockchain technologies. The blockchain must ensure trusted use of the sensor network’s infrastructure, which ultimately increases security [5]. However, blockchain is a rather complex and resource-intensive technology, in which trust is achieved using signatures, as in classical cryptography [6]. Deep learning authentication methods are another popular sensor security solution [7]. At the same time, researchers are looking for new signs of behavior assessment that would form the basis of authentication [8]. However, cryptographic and authentication methods cannot always ensure the complete security of a system.

Often the sensory system is located outside a controlled area, in natural conditions, or outdoors. The nodes of the system may be mobile, the architecture of the system can be periodically updated, and new nodes may be added [9]. Cryptographic and authentication methods cannot always ensure the complete security of such a system. In addition, many sensor systems are based on wireless networks that are themselves not physically secure [10]. Even if the nodes encrypt traffic and authenticate each other, this will not protect them from active attacks and other destructive influences and will not help to detect external influences [11].

One of the solutions to improve the security of sensor networks may be the modernization of the network’s underlying architecture, through improvement of its physical properties. Dodig et al. [12] propose modifying the architecture of Industry 4.0 systems to implement security solutions at the hardware level.

Stępień and Poniszewska-Marańda propose behavioral-based safety measures and algorithms for vehicular networks [13]. Their approach concentrates on vehicles assessing each other’s behavior. Various types of vehicle behavior are identified, which ultimately affects the level of confidence in it. In this case, it is necessary to comply with certain constraints, such as the distance to the neighboring car, and the number of neighbors. The authors claim that even if the number of intruder vehicles exceeds 50%, the system will still be able to detect them.

Jiang et al. [14] use statistical and machine learning methods to identify anomalies. At the same time, the authors analyze time series, classifying them into the following types: periodic, stationary, non-periodic, and non-stationary. Then, different schemes are applied to different classes of time series to detect anomalies. The authors declare that their semi-supervised anomaly detection framework method (called Tri-CAD) yields the best grading results.

Mittal et al. [15] analyze the security and performance of the most popular secure routing protocols, Low-Energy Adaptive Clustering Hierarchy (LEACH) [16], Energy-Efficient Sensor Routing (EESR), and Sub-Cluster LEACH. They show that Sub-LEACH with Large Margin Nearest Neighbor (LMNN) produces the best performance [17]. The authors also propose an intrusion detection system. They use data normalization and coding techniques for better processing.

Schneider et al. [18] use a ready-made collection of data to evaluate their method [19]. The authors analyze various methods for detecting attacks and anomalies, based on machine learning algorithms (Logistic Regression (LR), Support Vector Machine (SVM), Decision Tree (DT), Random Forest (RF), Artificial Neural Network (ANN), and k-Nearest Neighbor (KNN)), to protect against cybersecurity threats on the Internet of Things. In contrast to previous work on individual classifiers, they also analyze ensemble methods such as packing, boosting, and summation to improve the performance of their detection system [20,21,22,23]. The authors integrate feature selection, cross-validation, and multi-class classification for the cybersecurity field. Experimental results with an existing dataset of attacks demonstrate that the method can effectively identify cyberattacks.

Thus, we have seen that ensuring information security of sensor networks, and the detection of anomalies, is an important research topic. However, previous authors often focus on machine learning-style methods rather than identifying the basic classification criteria. In most studies, ready-made databases with already-known attacks are used for analysis and identification of anomalies. In our research, we focused on identifying the fundamental signs of an attack.

A key objective of our research is to study changes in cyber-physical parameters under the influence of an attack, both during normal functioning and with additional load. It is necessary to identify signs of an attack that could give a new insight into anomalies in the sensory system. In addition, the study should allow the creation of its own database, which can be considered authentic for artificial intelligence training. The database can then be used to test various classification methods.

Determination of attack signs and analysis of cyber-physical parameters as a new vector for assessing an attack was a priority task in our study. We also consider the issues of data normalization. By virtue of data normalization and detection of threshold values at the experimental stage, our method can be applied to any sensory system without the need for prior training. Most importantly, our approach allows nodes in a network to detect attacks on their neighbors, thus allowing them to work together to defend the entire network.

The rest of the paper is as follows. In Section 2, the architecture of the anomaly detection system is presented, its main modules are described, and the method for detecting anomalies is presented. Section 3 explains the experimental study, its results, and discussion, as well as attributes for classifying attacks. Finally, the conclusion and future work is discussed in Section 4.

## 2. Materials and Methods

### 2.1. Threat Model

The threat model in this study is based on the vulnerabilities of wireless communication channels between the sensor network nodes. The main threats are implemented using active attacks and can be considered as follows:

- Threat of deauthorization of an authorized wireless client. The threat is the ability to automatically disconnect a wireless access point from an authorized wireless client.
- Threat of unauthorized access to the system via wireless channels. The threat lies in the possibility of an intruder gaining access to the resources of the entire discredited information system through the wireless data transmission channels used in its composition.
- Threat of exploiting weaknesses in network/local communication protocols. The threat lies in the possibility of an intruder’s unauthorized access to information due to a destructive effect on the protocols of network/local data exchange in the system.
- Threat of remote consumption of sensor nodes resources. The threat is that an attacker can influence the consumed unit of energy resources (i.e., the amount of energy consumed per unit of time) by continuing to send packets to them, but also without allowing nodes to go into sleep mode.
- Threat of blocking wireless communication channels between nodes. The threat lies in the possibility of noise or blocking of one of the nodes participating in the network exchange, which leads to blocking of the communication line.

Let us consider the impact of different types of attacks on the cyber-physical parameters of the sensor node. The result of the analysis is shown in Table 1.

In the study described in this paper, scenarios of two active attacks were implemented. One of them is a SYN (Synchronize sequence numbers) flood attack, which was carried out using the netwox utility. The second is a deauthentication attack, which was carried out using the aireplay-ng utility. A SYN flood attack can be thought of as an availability attack and a host depletion attack. A deauthentication attack can be thought of as an accessibility attack.

### 2.2. Architecture of the Sensor Network Adaptive Protection System

In this subsection, we describe the assumed architecture of the adaptive protection system. The main capabilities that the system provides are:

- Detection of anomalies through analysis of the system node’s parameters;
- Timely notification of the operator and neighboring nodes about a possible incident; and
- Determination of the type of attack.

**Figure 1:**
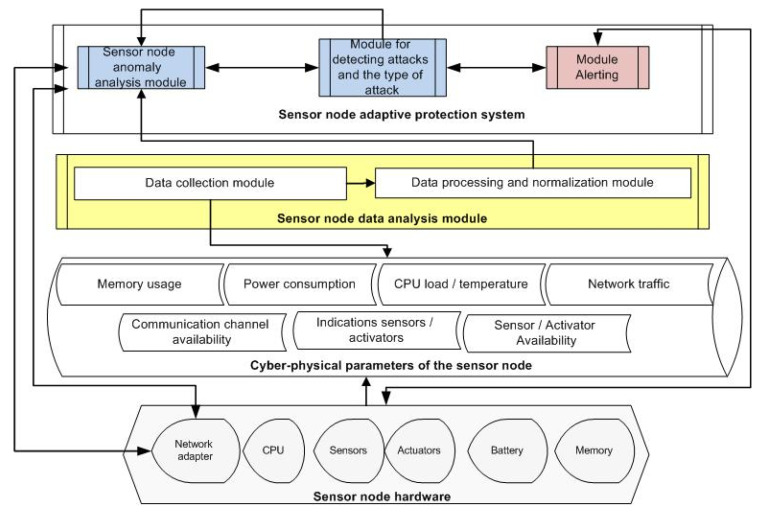
Block diagram of the adaptive protection system of the sensor node.

Figure 1 shows a general structural diagram of our protection system, including the main modules and subsystems, as well as their integration into the sensor system.

The subsystem for analyzing and collecting data from the sensor node collects information about changes in cyber-physical parameters. The subsystem receives data from the sensor node’s hardware. The key idea is that by analyzing cyber-physical parameters throughout the entire operation of the sensory system, it is possible to detect the presence of anomalies that could be signs of an active attack on the sensory system. Cyber-physical parameters are parameters that reflect changes in both software and physical components of the sensor node.

#### 2.2.1. Sensor Node Data Analysis Module

The module for analyzing data about the sensor node also includes a module for processing and normalizing data. Using raw data can be ineffective for several reasons. First, raw data requires more memory and processing power. Secondly, when processing raw data, false alarms or measurement inaccuracies can occur. Thus, in our approach, methods of probability theory were used to normalize data. Quantile diagrams were built to determine the types of probability distributions for each parameter. This helps to determine the distribution type of a random variable. The sensor node data analysis module collects information from hardware devices, sensors, and actuators about CPU utilization, power consumption, and memory resources, network traffic, etc. Then the data is normalized and transmitted to the sensor node anomaly analysis module. The advantages of using the data analysis subsystem are as follows:

- The ability to transfer collected and normalized data to other subsystems; and
- The ability to present the collected data in a simple format convenient for analysis.

#### 2.2.2. Cyber-Physical Parameters for Analysis

Figure 1 shows the use of cyber-physical parameters and hardware components of the sensor node by the adaptive protection system modules.

As can be seen, only the sensor node data analysis module accesses the hardware devices. It takes readings from sensors, computing resources, actuators and receives a set of cyber-physical parameters.

System modules work with processed data. Thus, the advantage of the sensor node data analysis subsystem is the use of one set of data for solving different protection tasks, as well as a decrease in the number of calls of software modules to hardware. Firstly, it provides greater reliability because the sensor node is controlled by a single process, while it is necessary to consider different processes in a single control system. If we talk about building a protection system, each process must be authorized, and its access to the hardware is controlled and recorded; otherwise, the system may fail or be compromised. Therefore, the fewer such calls, the easier it is to process these events from a security point of view. Secondly, the processing of software modules with prepared datasets speeds up the decision-making process, which increases the response time and, at the same time, the performance of the system. An attacker can carry out targeted attacks on certain cyber-physical parameters, or can carry out attacks that indirectly affect the physical properties of the system [24]. For example, there could be a direct attack on the depletion of a device’s battery, and an attack on a network overflow with false requests to connect to a node, which can be a sensor network node. In this case, the effects of both attacks can be similar. For example, both attacks can result in a greater computational load and drain the battery faster whereas an overflow attack on false requests also increases network traffic.

If we consider cyber-physical parameters in general terms, then it is quite difficult to track the difference in the impact of attacks on each of them. Many types of attacks are the same for different cyber-physical parameters. In addition, the evaluation of the parameter in a general form can give false positives. For example, an increase in network traffic may be associated with the need to transmit additional useful information, and not with an attack. However, if we consider the network traffic in more detail, and classify it by protocol types and direction, then the values can be more accurate. In addition, greater detail will allow identifying attack types in the future.

By considering which cyber-physical parameters changed their value and to what extent it becomes possible to determine not only the type or class of the attack but also to establish which attack was implemented on the system.

#### 2.2.3. Sensor Node Adaptive Protection System

This module is based on the detection of anomalies or abnormal behavior. As a consequence of their potentially destructive effect, the state of the sensory system changes from a normal state to an abnormal one. The main tasks performed are:

- Detecting anomalies and establishing a correlation between an anomaly and an attack; and
- Exchanging information with other modules in the protection system.

Anomalies and denials of service can occur not only as a result of a deliberate attack but also due to external natural influences or component failures associated with mistakes made in the design of the sensor system. To rule these out, it is necessary to study the process of diagnostics, forecasting, monitoring, and decision-making in real-time using data obtained from both a single sensor node and from the sensor system.

The module for detecting attacks and the type of attack on the sensor system classifies the detected anomaly as an attack and determines its type. The sensor node can be intercepted by an intruder and used as an attacking node or can harm the rest of the sensor system because of a technical, systemic failure. The type of attack is determined based on which cyber-physical parameters are affected and to what extent. The attack-detection module receives data from the anomaly-detection module and uses them for further analysis. The module for detecting attacks and the type of attack of the sensor node also interacts with the alert notification subsystem. Using this architecture, our research defines the set of rules for the further implementation of these modules.

#### 2.2.4. Alert Notification Module

This human-machine interface module is designed to send messages to the operator, who can monitor the operation of the sensor system and coordinate it. The module can be useful in mixed control when the operator gives commands, and the nodes are already in an autonomous mode to execute them.

If the system is configured so that nodes share tasks and act as a coordinated group, then this module will notify not only about anomalies detected for an individual node but about anomalies detected by neighboring nodes.

### 2.3. Method for Determining the Abnormal Activity of the Sensor System

#### 2.3.1. Technique for Processing and Normalizing Data

Here we describe our technique for processing the raw sensor data in order to identify abnormalities. A normal distribution smooths out the changes in the random variable, and the attack start time may not be fixed if the distribution is rebuilt every time interval [25]. If the range of values is significant, then the attack will be missed due to the fact that the standard deviation will rise sharply and may even exceed the expected value. After determining the initial conditions, a normal distribution is constructed:

(1) $$f(s)=\frac{1}{\sigma_{s}\sqrt{2\pi }}e^{-\frac{{\left(s-M_{s}\right)}^{2}}{2\sigma_{s}{}^{2}}},$$

(2) $$f(r)=\frac{1}{\sigma_{r}\sqrt{2\pi }}e^{-\frac{{\left(r-M_{r}\right)}^{2}}{2\sigma_{r}{}^{2}}},$$

where *f*(*s*) and *f*(*r*) are the normal distribution functions of a random variable (in this case, the number of packets sent and received, respectively) at specified time intervals; *M_s_* and *M_r_* are the mathematical expectations for sent and received packets, respectively, calculated in the same way for forwarded and dropped packets; and *σ_s_* and *σ_r_* are the standard deviations for the same parameters.

A Poisson distribution can be used to estimate the cyber-physical parameter of the CPU load [26]. The Poisson model describes a scheme of rare events: under certain assumptions about the nature of the process of occurrence of random events, the number of events that occurred over a fixed period or in a fixed region of space often obeys the Poisson distribution:

(3) $$P(K_{n})=\frac{\lambda^{K_{n}}}{K_{n}!}e^{-\lambda },$$

where *P* is the probability function of the distribution of a random variable according to the Poisson distribution; *K_n_* is the percentage of the total amount of processor time between *n* and *n*–1 that the processor spent on processing processes running in the kernel mode; *λ* is the mathematical expectation that is the average number of occurrences of the event of interest in a unit of time; and *e* is Euler’s number.

#### 2.3.2. The Method of Detecting Anomalies Based on Entropy in the Sensor System

Our analysis of the normalized cyber-physical parameters showed that the probability distribution of the victim and the normal state is often close to each other, but the abnormal state is significantly different [27]. To measure the difference between function distributions, the Kullback-Leibler entropy measure is used. The entropy of a random variable is a measure of its uncertainty; it is a measure of the amount of information required on average to describe a random variable. Relative entropy is a measure of the distance between two distributions [28]. In statistics, it appears as the expected logarithm of the likelihood ratio [27]. The relative entropy *D* (*p||q*) is the measure of inefficiency if the distribution is assumed to be *q* when the true distribution is equal to *p*. For example, if you know the true distribution *p* of a random variable, you can construct a code with an average description length *H* (*p*). If instead a code were used to distribute *q*, it would take on average *H* (*p*) *+ D* (*p||q*) bits to describe the random variable. Relative entropy was first determined by Kullback and Leibler [29,30].

1. Determination of the measure of entropy for the cyber-physical parameter (the level of CPU load):

(4) $$KL(PK_{ni}||PK_{nj})=\sum PK_{ni}(x)|\ln\frac{PK_{ni}(x)}{PK_{nj}(x)}dx=D_{ij}$$

(5) $$KL(PK_{nj}||PK_{ni})=\sum PK_{nj}(x)\ln\frac{PK_{nj}(x)}{PK_{ni}(x)}dx=D_{ji}$$

where *PK_ni_* is the function of the probability distribution of the CPU workload of a random variable of the sensor node *i* at the current time interval; *PK_nj_* is the function of the probability distribution of the CPU workload of a random variable of the sensor node *j* at the current time interval; *D_ij_* is the degree of deviation of the distributions of node *i* from node *j*; and *D_ji_* is the degree of deviation of the distributions of node *j* from node *i*;

2. Determination of the measure of entropy for the cyber-physical parameter (network traffic):

(6) $$KL(f{\left(s,r\right)}_{ni}||f{\left(s,r\right)}_{nj})=\int f{\left(s,r\right)}_{ni}\ln\frac{f{\left(s,r\right)}_{ni}}{f{\left(s,r\right)}_{nj}}d(s,r)=DL_{ij}$$

(7) $$KL(f{\left(s,r\right)}_{nj}||f{\left(s,r\right)}_{ni})=\int f{\left(s,r\right)}_{nj}\ln\frac{f{\left(s,r\right)}_{nj}}{f{\left(s,r\right)}_{ni}}d(s,r)=DL_{ji}$$

where *f*(*s*,*r*)*_ni_* is the function of normal distribution of network traffic of the sensor node *i* for on the current time interval; *f*(*s*,*r*)*_nj_* is the function of normal distribution of network traffic of a random variable *j* on the current time interval; *DL_ij_* is the degree of deviation of the distributions of node *i* from node *j*; and *DL_ji_* is the degree of deviation of the distributions of node *j* from node *i*.

Thus, using this method, it is possible to determine to what extent the behavior of a given node *i* differs from a neighboring node *j* and to identify anomalies.

## 3. Results and Discussion

Based on the protection system architecture and data analysis equations above, we conducted practical experiments to both validate and configure the anomaly detection process. The main objectives of the experimental study were as follows:

- confirmation of the effectiveness of the method for detecting anomalies of the sensor system;
- collecting data to form a data set for training a neural network, to classify attacks; and
- analysis of the boundaries of divergence values for making decisions about the presence of anomalies and attacks in the sensory system.

The experimental study was carried out using a test bench developed and presented by the authors earlier [31]. The test bench is a set of single-board computers with a Linux-based operating system installed. The stand includes 4 nodes that exchange useful information according to a given algorithm, while a wireless mesh network is created between the nodes.

The experimental study was carried out in four directions:

- Recording the normal operation of the sensor system, when the nodes exchange information according to a given algorithm using the User Datagram Protocol (UDP) [32], and when the Optimized Link State Routing (OLSR) protocol [33] is used between the nodes. In this case, there is no additional effect on the sensory system.
- Adding a payload to normal node operation. The Internet Control Message Protocol (ICMP) was used as the payload and the request/response messages were sent to the neighboring node.
- A denial-of-service attack aimed at overloading a node. To implement this scenario, a SYN flood attack was used, the victim’s open port was attacked, in this case, Port 22 [34]. During the attack, the victim node received many connection requests, because of which the message queue overflowed, and the node was blocked, while the network remained available.
- A denial-of-service attack aimed at blocking a channel. To implement this scenario, a deauthentication attack was used. When one of the nodes was blocked and its connection with other corners was lost, packets were not transmitted between neighboring nodes [35]. At the same time, the work of the node itself was not blocked, but it simply could not receive a response to the messages transmitted to it.

### 3.1. Analysis of Node Behavior during Normal Operation

**Figure 2:**
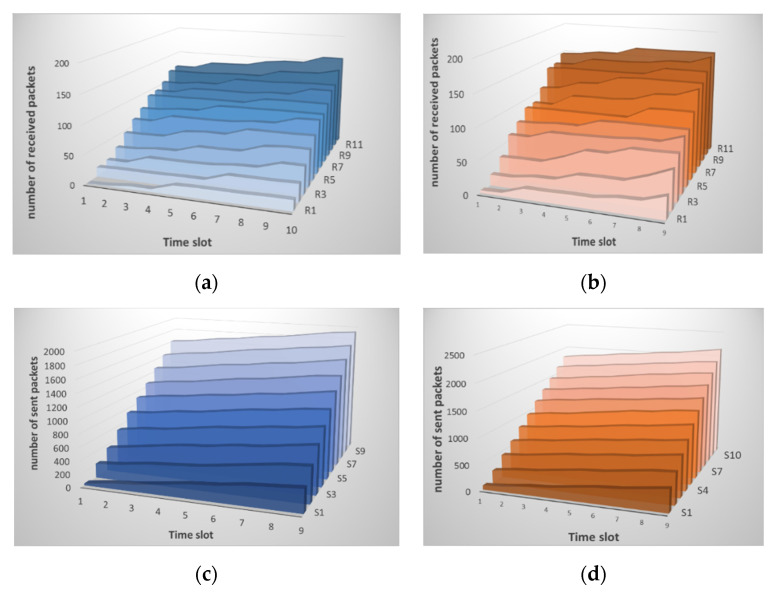
Changes in the traffic pattern when analyzing raw data on: (**a**) received packets of node *i* and (**b**) packets sent from node *i*; (**c**) received packets from node *j* and (**d**) sent packets from node *j* during normal operation of the sensor network.

As mentioned earlier, the nodes exchange messages according to a previously defined algorithm. They send packets based on their calculations, not monotonously. Therefore, as can be seen from Figure 2, the traffic picture does not look straightforward. Small changes are observed from one time series to another. In general, the number of transmitted and received packets grows over time.

**Figure 3:**
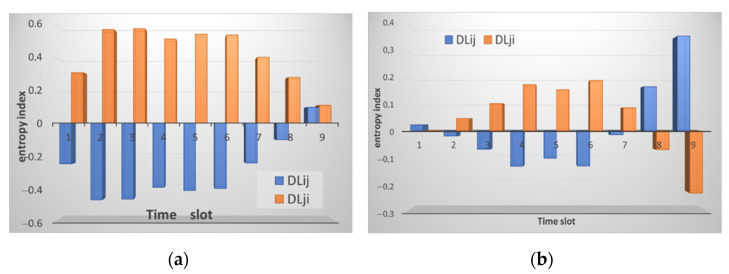
The result of calculating the entropy for: (**a**) received packets and (**b**) sent packets by node *i* relative to node *j* (marked in blue) and node *j* relative to node *i* (marked in orange).

After the data analysis module receives the traffic information, it normalizes it, and the entropy is calculated, as shown in Section 2.2 above. The result of calculating entropy by neighboring nodes is shown in Figure 3. Figure 3a shows the result of calculating entropy for incoming traffic. Small deviations are observed, and the peak value reaches 0.5. This situation is normal and does not indicate an attack. Figure 3b shows the computation result for received packets.

As can be seen from the figures, the entropy values for the received packets are lower than for the sent ones. This situation correlates with the traffic picture obtained for the raw data. Figure 2 shows that the traffic changes are slightly larger for received packets. Although it can be seen from Figure 2b that the number of sent packets themselves is much larger than the number of received ones. That is, the method allows us to detect deviations regardless of the number of packets, namely, on the behavior of the node when sending/receiving them. In this case, there are no false alarms, and no thresholds are exceeded. It is important to note that, according to this method, the nodes analyze not themselves, but each other. Figure 3 shows that there is practically no difference between the sent packets of the two nodes, the graphs are uniform, although the number of packets is different. The graphs of received packets are more different but not significantly.

**Figure 4:**
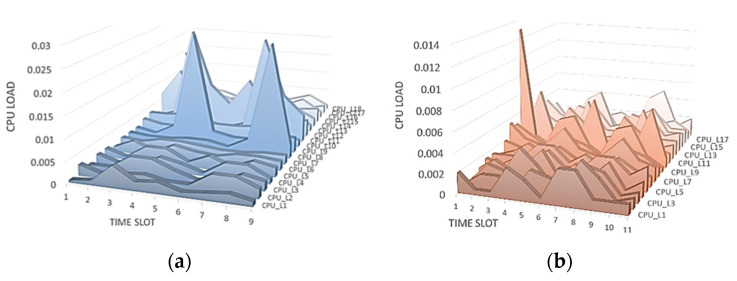
Changing the CPU load when analyzing raw data: (**a**) for node *i*; and (**b**) for node *j*.

Next, we considered changing the CPU load parameter. In this study, the load is determined not as a percentage, but in processor ticks for a fraction of a second. As can be seen from Figure 4, at first glance, the CPU load parameter for nodes *i* and *j* is different.

**Figure 5:**
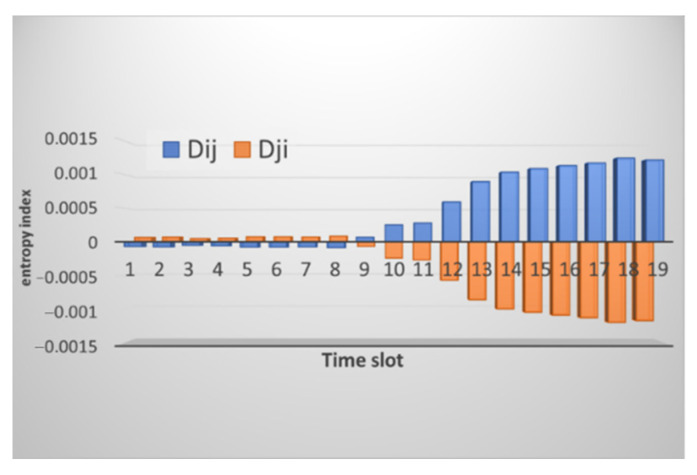
Result of calculating the entropy for the level of CPU utilization by node *i* relative to node *j* (marked in blue) and by node *j* relative to node *i* (marked in orange).

At an in-depth analysis, we can see large differences in values, and no overall picture is uniform. The CPU load cannot be the same at different intervals since, by its very nature, this parameter changes abruptly. The result of calculating the entropy for node *i* for node *j* is presented in Figure 5. We can see that a small difference in values is observed on time intervals starting from the 10th, which correlates with the raw data.

### 3.2. Analysis of Node Behavior with Additional Payload

**Figure 6:**
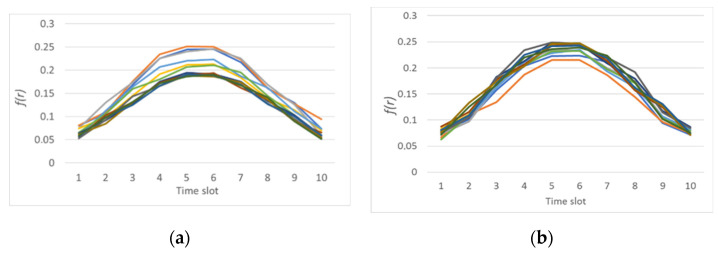
The result of calculating the normal distribution function for incoming traffic of: (**a**) node *i* with additional payload; and (**b**) node *j* in normal mode.

When providing an additional payload that is not an attack, some changes were observed in the sensory system. An additional payload was that the node sent echo requests using the ping command. Figure 6 shows the result of calculating the normal distribution function for the incoming traffic of a node on which an additional payload is rendered and a node that is operating normally.

**Figure 7:**
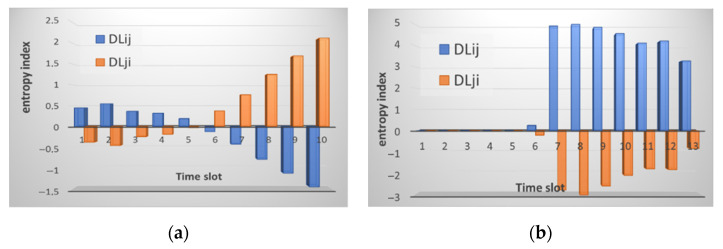
The result of calculating the entropy for: (**a**) received packets; and (**b**) sent packets by node *i* relative to node *j* (marked in blue) and by node *j* relative to node *i* (marked in orange) with additional payload.

Figure 6 shows that for the incoming traffic of node *i*, the normal distribution function is shifted down. This offset will affect the entropy calculation. The result of calculating the entropy is shown in Figure 7. It shows that there is a slight increase in the values for outgoing traffic.

When the value does not exceed two then, conditionally, the network operates in normal mode. In this case, a slight excess is noticeable. This excess of the entropy value can be interpreted as a change in the operating mode or additional load. As Figure 7 reveals, after increasing the traffic value, then a decrease in entropy is observed. That means that the system comes to a steady state.

**Figure 8:**
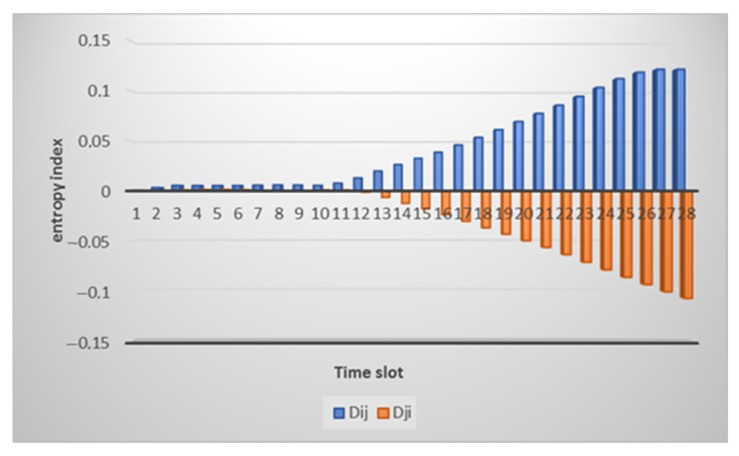
The result of calculating the entropy for the level of CPU utilization by node *i* relative to node *j* (marked in blue) and by node *j* relative to node *i* (marked in orange) with an additional payload.

Figure 8 shows the entropy result for the CPU utilization. The graph is very similar to the result of calculating the entropy for the normal state. Entropy values reach 0.1. However, this value is far from the 0.5 threshold. This suggests that some additional activity is present, but not abnormal.

Thus, only one parameter out of three showed changes exceeding the threshold, the outgoing traffic. The rest of the parameters were within the normal range, although they increased slightly.

### 3.3. Analysis of Node Behavior in a Denial of Service Attack—SYN Flood

**Figure 9:**
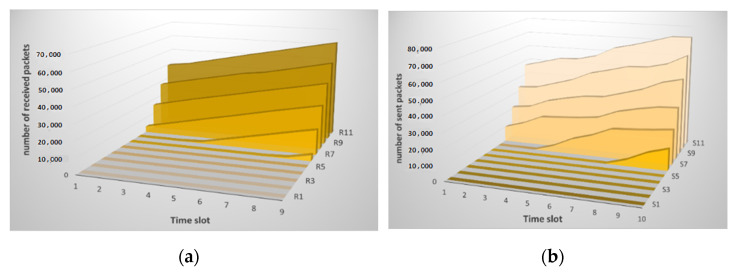
Changes in the traffic pattern when analyzing raw data on: (**a**) received packets from node *i*; and (**b**) sent packets from node *i* under conditions of a SYN flood attack.

In our study, the SYN flood attack did not start immediately but three minutes after the start of the experiment. Until then, the sensor system functions normally. Figure 9 shows that changes begin in the fifth time series for both inbound and outbound traffic.

**Figure 10:**
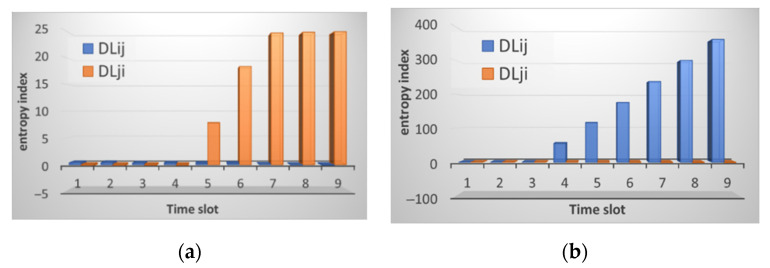
The result of calculating the entropy for: (**a**) received packets; and (**b**) sent packets by node *i* relative to node *j* (marked in blue) and node *j* relative to node *i* (marked in orange) under conditions of a SYN flood attack.

The changes are accompanied not only by a sharp increase in the number of packets but also by a change in the traffic picture. If you compare the traffic pattern during an attack and during normal operation, you will notice that the graphs become more straightforward. This is because the traffic associated with the attack overlaps the useful one and other “noise” in the traffic. Figure 10 shows the result of calculating the entropy for incoming and outgoing traffic by node *i* relative to node *j* and vice versa.

**Figure 11:**
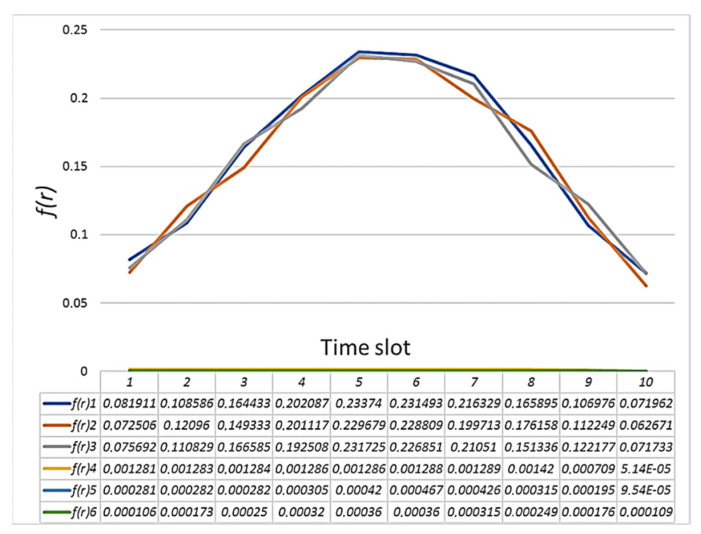
The result of calculating the normal distribution function for the incoming traffic of node *i* under conditions of a SYN flood attack.

The figures show a significant increase in the entropy index. In one case for incoming traffic, node *j* manages to identify the anomaly, and for incoming traffic, node *i* does so. Figure 11 shows the result of calculating the normal distribution function for incoming packets of the attacked node. At the same time, the graying of three intervals looks like a straight line, but in fact it is not. The values of the normal distribution function for the last three intervals during the attack are so small that they are difficult to see on the graph. When calculating the entropy, the results of calculating the normal distribution function are compared. Precisely because the values of the normal distribution during the attack become too small, node *i* when comparing them with node *j*, does not identify the anomaly, while in the opposite direction everything works, and node *j* detects an anomaly.

**Figure 12:**
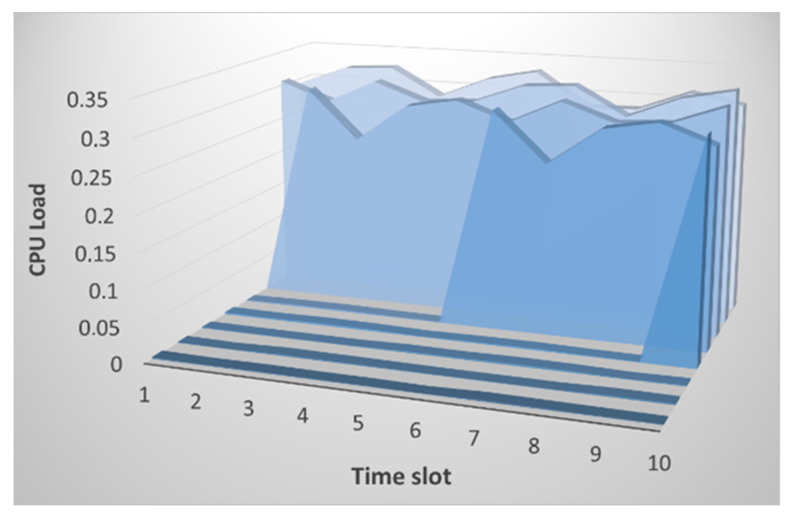
Changing the CPU load when analyzing raw data for node *i* under conditions of a SYN flood attack.

The CPU utilization is also significantly affected by the attack. Figure 12 shows the raw CPU utilization data. At first, a sharp jump in values is observed, and then the situation becomes like that without an attack, only for higher values.

**Figure 13:**
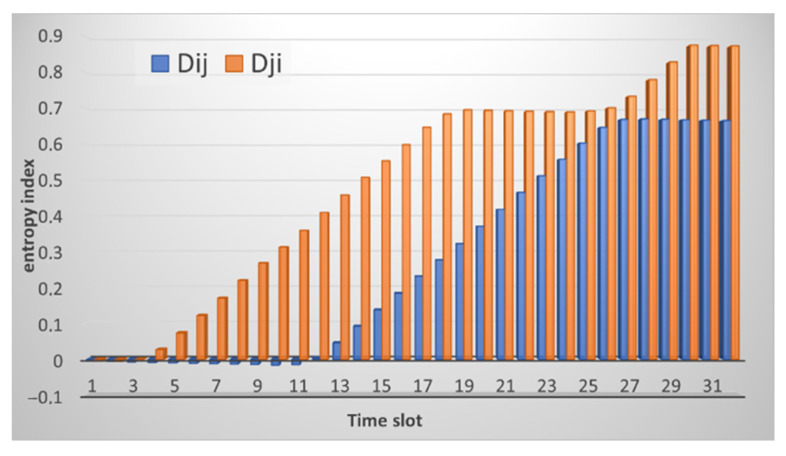
The result of calculating the entropy for CPU load by node *i* relative to node *j* (marked in blue) and by node *j* relative to node *i* (marked in orange) under conditions of a SYN flood attack.

Now we analyze the change in CPU utilization during the attack and without it; node *i* is under attack, but node *j* is not. Figure 13 compares their assessment of the load level with each other.

Figure 13 shows that, at first, there is an increase in the value of the divergence which is fixed by both nodes, and then the value remains at the same level. This is due precisely to the fact that no more abrupt changes in the CPU load are observed. This method allows us to record the fact of a change in state and the degree of these changes, which is fundamental. Estimating thresholds for CPU utilization is easier because the closer the value is to one, the more likely an attack is to occur. In this case, the value reaches 0.9. However, increasing values to 0.5 can also be viewed as a change of state.

### 3.4. Analysis of Node Behavior in a Denial of Service Attack—Deauthentication

**Figure 14:**
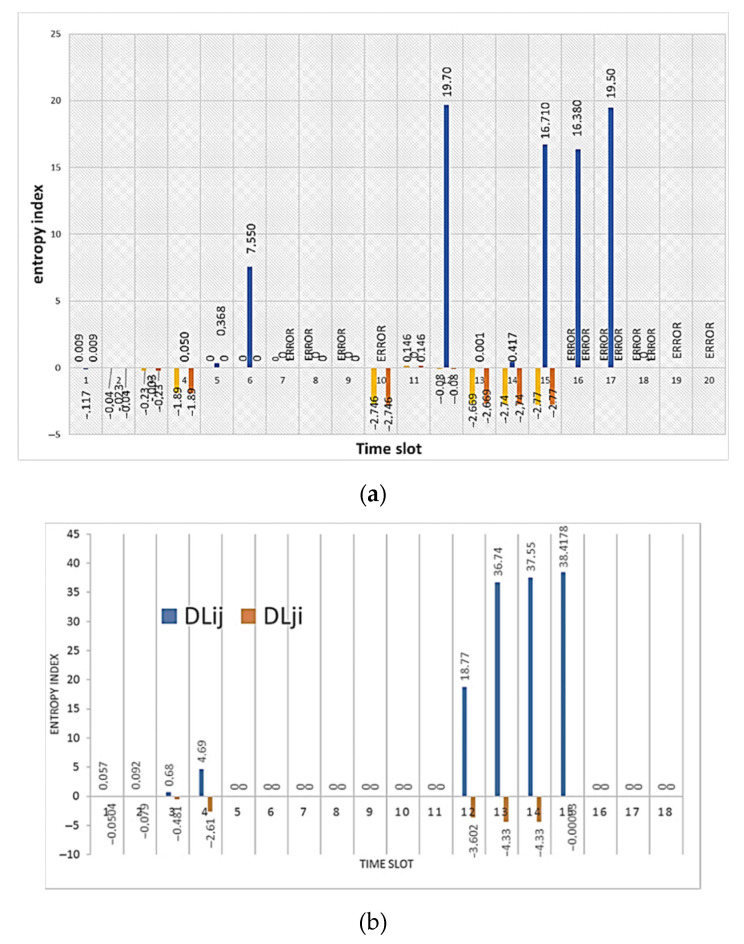
The result of calculating the entropy for: (**a**) received packets; and (**b**) packets sent by node *i* relative to node *j* (marked in blue) and by node *j* relative to node *i* (marked in orange) under conditions of a deauthentication attack.

During a deauthentication attack, the connection is broken, and the sensor node cannot fully exchange data with neighboring nodes. Figure 14 shows the result of calculating the entropy for node *i*, which was attacked. It can be seen from the figures that not only is an increase in the entropy value observed, but also there are no values for some time intervals. These gaps occur due to the fact that the entropy formula contains the logarithm of the quotient and the normal distribution function for an attacked node can take zero values, but division by zero is impossible, of course.

**Figure 15:**
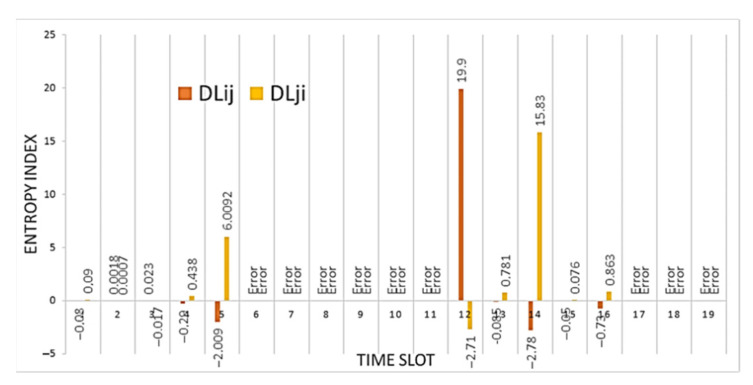
The result of calculating the entropy for received packets by node *i* relative to node *j* (marked in yellow) and by node *j* relative to node *i* (marked in orange) under conditions of a deauthentication attack.

In general, it can be noted that the attack is readily identifiable for node *i*; the entropy values are quite high. Let us analyze the situation when node *j* is also influenced. This situation can occur if nodes are adjacent and, due to blocking of node *i*, node *j* also cannot exchange messages, like a Black Hole attack. The calculation result is shown in Figure 15.

**Figure 16:**
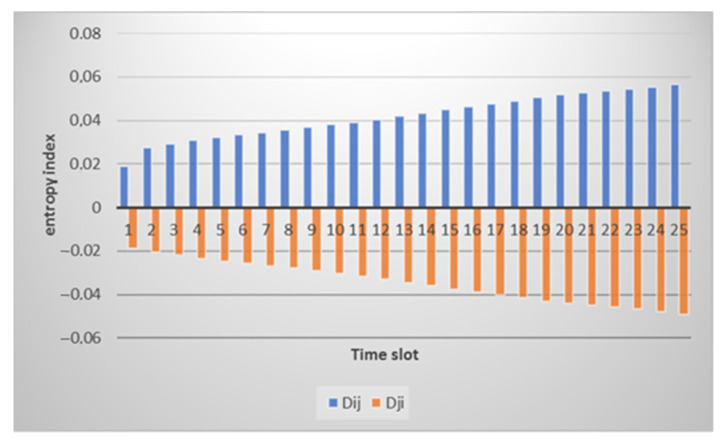
The result of calculating the entropy for CPU load by node *i* relative to node *j* (marked in blue) and by node *j* relative to node *i* (marked in orange) by the impact of a deauthentication attack.

Figure 15 shows that even though both nodes are susceptible to attacks, they both detect an anomaly, and this is evidenced by high entropy values. The attack affects each node differently, so changing the parameters is also different, which is recorded by our method. When it comes to changing the CPU load, the situation is reversed. The CPU utilization is reduced during the attack. This is because the node is not wasting power for forwarding packets. Therefore, as can be seen from Figure 16, the entropy level for the CPU load is low.

Based on the results of our experimental study, the following conclusions can be drawn about the effectiveness of our approach.

Firstly, as stated earlier, each attack has a different effect on the parameters. In this study, only three parameters were considered: incoming and outgoing traffic, and the level of CPU utilization. Nevertheless, even according to just these three parameters, it is possible to determine the type of attack and classify it.

Secondly, the entropy-based method allows us to capture the difference between the behavior of nodes by increasing the values. Thirdly, depending on the type of probability distribution that is used to normalize the parameter, the thresholds change. If the Poisson distribution is used, then the upper limit of the value is one, and anything between 0.5 and one indicates an attack. If the normal distribution is used for normalization, then there is no upper bound, and the values can be very high if an attack occurs.

Therefore, the threshold value can be set differently. As shown by numerous experiments, some of which are presented in this article, the attack is characterized by an increase in the entropy value of more than 10. If the values increase up to 10, then this may indicate a change in activity or minor changes in the sensory system. Table 2 shows the result of the analysis of the obtained values.

Thus, it can be seen from this table that if there is a sharp increase in all three parameters, which is detected by at least one of the neighboring nodes, then a SYN flood attack is carried out. If an insignificant increase in one of the parameters is observed, then a change in the operating mode or additional load on the node may occur. If there is an increase in entropy for traffic as well as erroneous values being observed, then a deauthentication attack or a break in the communication channel has occurred. If this situation is observed for both nodes, then a Black Hole attack is likely. In this case, the CPU load does not change significantly.

We also have estimated the autonomous operating time of the device considering the Raspberry Pi 3B with the installed Raspbian operating system. Its consumption current was about 200–210 mA without additional load, 210–220 mA when the program was running, and the peak value of 230–240 mA was reached during the formation and sending of packets to the neighbors. Thus, we have assumed a maximum consumption current of 240 mA for our device. In addition, a battery with a capacity of 4000 mAh was available for the Raspberry Pi 3B, so the autonomous operating time of the device has been estimated as over 16.6 h.

## 4. Conclusions

This article addressed the issues of detecting anomalies in sensor networks. Existing anomaly detection systems usually use machine learning methods. At the same time, previous authors mainly focus on the development and modification of classification methods and algorithms, proving their effectiveness. However, the issues of detection metrics are often omitted and not considered. Many articles present the use of off-the-shelf signature sets and datasets for classification and training. However, there remains a question about the validity of data for training from open sources, as well as the applicability of learning outcomes to ready-made systems. Some other works consider specific behavioral scenarios of nodes, especially for mobile nodes of the network, as signs of trust [36,37,38].

Our research instead focused on the assessment of cyber-physical parameters and their changes under various operating modes of the system. The measure of entropy and normalization of raw data allows data to be unified and evaluated in terms of detecting anomalies. This study examined in detail the changes in three parameters for four scenarios. Already, at this stage, using only three parameters, it is possible to determine the difference between attacks and variants of normal behavior. It is possible to evaluate not only the fact of a parameter change but also evaluate the degree of its change. At the same time, each node of the sensor network can compare its changes with the changes of neighboring nodes. Most importantly, using our method, nodes can analyze each other for evidence of attacks.

As an extension of the method, we can add a comparison of the node with itself and evaluate the changes in its own parameters over time. It is also planned to increase the number of estimated cyber-physical parameters that can be affected by an attack. In addition, it is possible to increase the number of attacks for evaluation too. Already, at this stage, anomalous behavior is unambiguously detected. Even if a specific attack scenario is unknown, it is possible to unambiguously establish which properties and which structural characteristics of the sensory system it affects.

Further research plans to use and compare intelligent methods to classify attacks based on the collected dataset, testing the method for errors of the first and second kind, as well as testing new attack scenarios and options for normal behavior for the sensory system. It is also necessary to test the method for various types of sensory systems.

## Figures and Tables

**Table 1:** Influence of various types of attacks on the cyber-physical parameters of the sensor system.

No.	Cyber-Physical Parameter	Attack Type
**1**	Memory usage	resource exhaustion attack, availability attack
**2**	Power consumption	resource exhaustion attack, availability attack
**3**	Communication channel	accessibility attack, access attack, integrity attack, confidentiality attack
**4**	CPU load	resource exhaustion attack, availability attack
**5**	CPU temperature	resource exhaustion attack, availability attack
**6**	Network traffic	integrity attack, privacy attack, accessibility attack
**7**	Sensor/Activator Availability	integrity attack, availability attack, access attack

**Table 2:** Analysis of the ability to classify attacks by our anomaly detection method.

Activity Type	Entropy of Incoming Traffic	Entropy of Outgoing Traffic	Entropy of CPU Load	Note
Normal operation	no increases	no increases	no increases	Received packets: −1 < *DL_ij_* < 1; −1 < *DL_ji_* < 1 Sent packets: −0.5 <*DL_ij_* < 0.5; −0.5 <*DL_ji_* <0.5 CPU load: −0.02 < *D_ij_* < 0; 0 < *D_ji_* < 0.02
Payload	in the normal range	increases	in the normal range	Received packets: −1 < *DL_ij_* < 1; −1 < *DL_ji_* < 1 Sent packets: −0.5 < *DL_ij_* < 0.5; −0.5 < *DL_ji_* < 0.5 CPU load: −0.02 < *D_ij_* < 0; 0 < *D_ji_* < 0.02
SYN flood attack	significant increase	significant increase	significant increase	Received packets: 0 < *DL_ij_* < 1; 7 < *DL_ji_* < 25 Sent packets: 10 < *DL_ij_* < 400; −0.5 < *DL_ji_* < 1 CPU load: −0.01 < *D_ij_* < 0; 0 < *D_ji_* < 0.9
Deauthentication	significant increase	significant increase	in the normal range	Received packets: 0 < *DL_ij_* < 20; −3 < *DL_ji_* < 0 Sent packets: 0 < *DL_ij_* < 40; −5 < *DL_ji_* < 1 CPU load: −0.06 < *D_ij_* < 0; 0 < *D_ji_* < 0.06

## Data Availability

Data sharing not applicable.
